# A novel statistical method for long-term coronavirus modelling

**DOI:** 10.12688/f1000research.125924.1

**Published:** 2022-11-10

**Authors:** Oleg Gaidai, Ping Yan, Yihan Xing, JingXiang Xu, Yu Wu

**Affiliations:** 1 Engineering Research Center of Marine Renewable Energy, Shanghai Ocean University, Shanghai, China; 2 Department of Mechanical and Structural Engineering and Materials Science, University of Stavanger, Stavanger, Norway

**Keywords:** COVID-19, Epidemic outbreak, Probability forecast, Public health, Mathematical biology

## Abstract

**Background**: Novel coronavirus disease has been recently a concern for worldwide public health. To determine epidemic rate probability at any time in any region of interest, one needs efficient bio-system reliability approach, particularly suitable for multi-regional environmental and health systems, observed over a sufficient period of time, resulting in a reliable long-term forecast of novel coronavirus infection rate. Traditional statistical methods dealing with temporal observations of multi-regional processes do not have the multi-dimensionality advantage, that suggested methodology offers, namely dealing efficiently with multiple regions at the same time and accounting for cross-correlations between different regional observations.

**Methods**: Modern multi-dimensional novel statistical method was directly applied to raw clinical data, able to deal with territorial mapping. Novel reliability method based on statistical extreme value theory has been suggested to deal with challenging epidemic forecast. Authors used MATLAB optimization software.

**Results**: This paper described a novel bio-system reliability approach, particularly suitable for multi-country environmental and health systems, observed over a sufficient period of time, resulting in a reliable long-term forecast of extreme novel coronavirus death rate probability. Namely, accurate maximum recorded patient numbers are predicted for the years to come for the analyzed provinces.

**Conclusions**: The suggested method performed well by supplying not only an estimate but 95% confidence interval as well. Note that suggested methodology is not limited to any specific epidemics or any specific terrain, namely its truly general. The only assumption and limitation is bio-system stationarity, alternatively trend analysis should be performed first. The suggested methodology can be used in various public health applications, based on their clinical survey data.

## Introduction

Statistical aspects of coronavirus disease 2019 (COVID-19) and other similar recent epidemics were receiving much attention in the modern research community. ^1^ Generally, it is quite challenging to calculate realistic biological system reliability factors and outbreak probabilities under actual epidemic conditions by using conventional theoretical statistical methods. ^1^^–^^14^ The latter is usually due to many degrees of system freedom and random variables governing dynamic biological systems, spread over extensive terrain. In principle, the reliability of a complex biological system may be accurately estimated straightforwardly by having enough measurements or by direct Monte Carlo simulations. ^10^ For COVID-19, however, the only available observation numbers are limited as the observations are only available from the beginning of the year 2020 up to now. Motivated by the latter argument, the authors have introduced a novel reliability method for biological and health systems to predict and manage epidemic outbreaks more accurately, this study was focused on COVID-19 epidemics in northern China, with focus on cross-correlations between different provinces within same climatic zone. For other studies related to statistical variations per country see, ^15^ where spatial lags were addressed. China was chosen to test the methodology proposed in this study, because of its COVID-19 origin and extensive health observations and related research available online. ^15^^–^^29^

Statistical modelling of lifetime data or extreme value theory (EVT) is widespread in medicine and engineering. For example, Gumbel used EVT to estimate the demographic of various populations in. ^30^ Similarly, in Ref. 31, the author builds on this EVT theory to determine the fitness effect using a Beta-Burr distribution. While in Ref. 32, the author discusses using a bivariate logistic regression model, which was then used to access multiple sclerosis patients with walking disabilities and in a cognitive experiment for visual recognition. Finally, Ref. 33 is a paper of relevance, which used EVT to estimate the probability of an influenza outbreak in China. The author demonstrated a forecasting prediction potential amid the epidemic in this paper. While in Ref. 34 similarly used EVT to predict and detect anomalies of influenza epidemics.

In this paper epidemic outbreak is viewed as unexpected incident that may occur at any province of a given country at any time, therefore spatial spread is accounted for. Moreover, a specific non-dimensional factor λ is introduced to predict the latter epidemic risk at any time and any place.

Biological systems are subjected to ergodic environmental influences. The other alternative is to view the process as being dependent on specific environmental parameters whose variation in time may be modelled as an ergodic process on its own. The incidence data of COVID-19 in all provinces of the People's Republic of China (PRC) from February 2020 until today were retrieved from the official public PRC health website, for simplicity only northern provinces were selected for this study. As this dataset is organized by province (more than 30 provinces in China), the biological system under consideration can be regarded as a multi-degree of freedom (MDOF) dynamic system with highly inter-correlated regional components/dimensions. Some recent studies have already used statistical tools to predict COVID-19 development, for linear log model see Ref. 9, these studies however did not address fully dynamic space-time dynamic bio-system as this study does.

Note that while this study aims at reducing risk of future epidemic outbreaks by predicting them, it is solely focused on daily registered patient numbers and not on symptoms themselves. For long-lasting COVID-19 symptoms, the so-called “long COVID”, and its risk factors and whether it is possible to predict a protracted course early in the disease, see Ref. 35, for mortality research see Ref. 36.

## Methods

This section presents novel bio-system reliability method, presently invented by authors and previously not existing. Advocated methodology used generalized extreme value (GEV) theory as a basis for its buildup. For numerical part authors used commercial software MATLAB, (Mathworks, V 8.6), namely its optimization routines, otherwise authors have used extrapolation code available from ACER. Only the code available from ACER was used to complete all sections of the methods – both numerical part as well as final extrapolation.

The novel method introduces MDOF (multi-degree of freedom) health response vector process $\;R\left(t\right)=\left(X\left(t\right),Y\left(t\right),Z\left(t\right),\ldots \right)$ that was measured over a sufficiently long period of time $\;\left(0,T\right)$ . Unidimensional global maxima over the entire time span $\left(0,T\right)$ denoted as $X_{T}^{\max}=\max_{0\leq t\leq T}X\left(t\right)$ , $Y_{T}^{\max}=\max_{0\leq t\leq T}Y\left(t\right)$ , $Z_{T}^{\max}=\max_{0\leq t\leq T}Z\left(t\right),\ldots$ .

Let $X_{1},\ldots ,X_{N_{X}}$ be time local maxima of the process $X\left(t\right)$ consequent in time, recorded at discrete time instants $t_{1}^{X}<\ldots <t_{N_{X}}^{X}$ that are monotonously increasing in $\;\left(0,T\right)$ . A similar definition follows ^1^ for other MDOF response components $Y\left(t\right),Z\left(t\right),\ldots$ with $Y_{1},\ldots ,Y_{N_{Y}};$ $Z_{1},\ldots ,Z_{N_{Z}}$ and so on. For simplicity, all $R\left(t\right)$ components, and therefore its maxima are assumed to be non-negative.

The target is to estimate system failure probability, in other words the probability of exceedance

$$1-P=\text{Prob}\left(X_{T}^{\max}>\eta_{X}\cup Y_{T}^{\max}>\eta_{Y}\cup Z_{T}^{\max}>\eta_{Z}\cup \ldots \right)$$ (1)

where $P={∭}_{\left(0,0,0,,\ldots \right)}^{\left(\eta_{X},\eta_{Y},\eta_{Z},\ldots \right)}p_{X_{T}^{\max},Y_{T}^{\max},Z_{T}^{\max},\ldots }\left(X_{T}^{\max},Y_{T}^{\max},Z_{T}^{\max},\ldots \right)dX_{T}^{\max}dY_{N_{Y}}^{\max}dZ_{N_{z}}^{\max}\ldots$ being the probability of non-exceedance for critical values of response components $\;\eta_{X}$ , $\eta_{Y}$ , $\eta_{Z}$ ,…; ∪ denotes logical unity operation «or»; and $p_{X_{T}^{\max},Y_{T}^{\max},Z_{T}^{\max},\ldots }$ being joint probability density of the global maxima over the entire period $\left(0,T\right)$ . However, it is not feasible to estimate the latter joint probability distribution directly due to its high dimensionality and available dataset limitations.

More specifically, the moment when either $X\left(t\right)$ exceeds $\eta_{X}$ , or $Y\left(t\right)$ exceeds $\eta_{Y}$ , or $Z\left(t\right)$ exceeds $\eta_{Z}$ , and so on, the system is regarded as immediately failed. Fixed failure levels $\eta_{X}$ , $\eta_{Y}$ , $\eta_{Z}$ ,…are, of course, individual for each unidimensional response component of $R\left(t\right)$ . $X_{N_{X}}^{\max}=\max\;\left\{X_{j};j=1,\ldots ,N_{X}\right\}=X_{T}^{\max}$ , $Y_{N_{Y}}^{\max}=\max\;\left\{Y_{j};j=1,\ldots ,N_{Y}\right\}=Y_{T}^{\max}$ , $\;Z_{N_{z}}^{\max}=\max\;\left\{Z_{j};j=1,\ldots ,N_{Z}\right\}=Z_{T}^{\max}$ , and so on.

**Figure 1.**
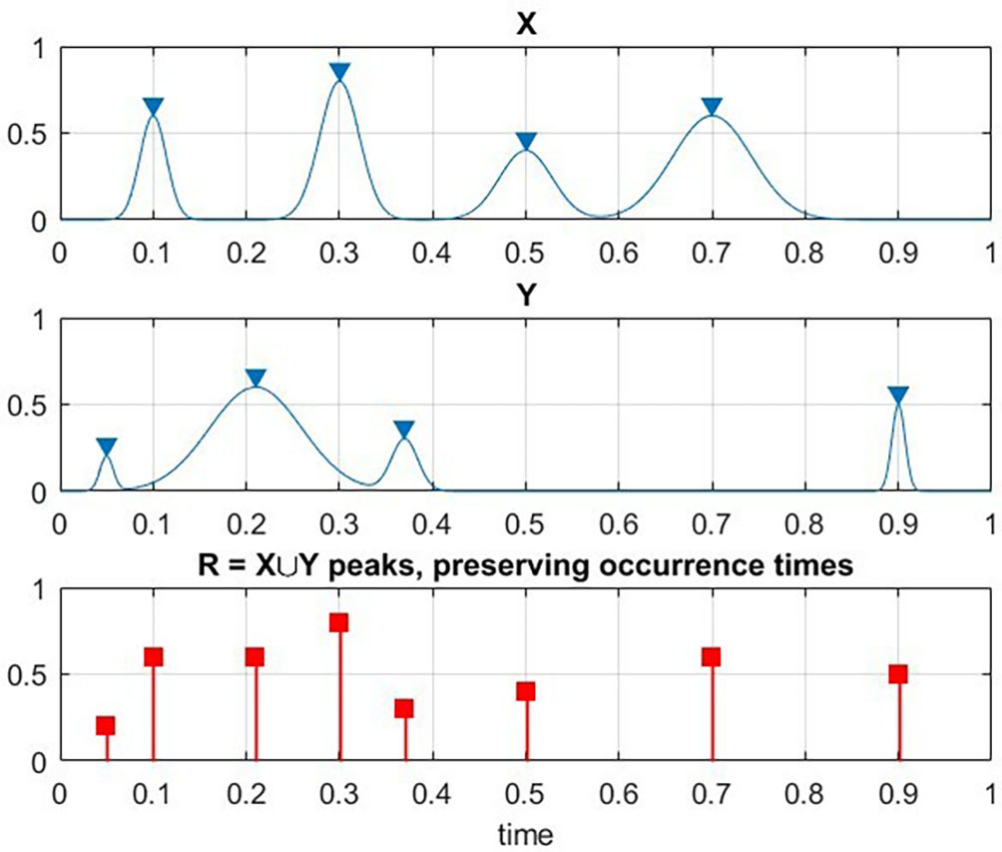
Illustration on how two exemplary processes X and Y are combined into new synthetic vector R.

Now, the local maxima time instants $\left[t_{1}^{X}<\cdots <t_{N_{X}}^{X};t_{1}^{Y}<\cdots <t_{N_{Y}}^{Y};t_{1}^{Z}<\cdots <t_{N_{Z}}^{Z}\right]$ are sorted in monotonously non-decreasing order into one single merged time vector $t_{1}\leq \cdots \leq t_{N}$ . Note that $t_{N}=\max\;\left\{t_{N_{X}}^{X},t_{N_{Y}}^{Y},t_{N_{Z}}^{Z},\cdots \right\}$ , $N=N_{X}+N_{Y}+N_{Z}+\ldots$ . In this case $t_{j}$ represents local maxima of one of MDOF structural response components either $X\left(t\right)$ or $Y\left(t\right)$ , or $Z\left(t\right)$ and so on. That means that having $R\left(t\right)$ time record, one just needs continuously and simultaneously screen for unidimensional response component local maxima and record its exceedance of MDOF limit vector $\left(\;\eta_{X},\eta_{Y},\eta_{Z},\ldots \right)$ in any of its components X,Y,Z,… Local unidimensional response component maxima are merged into one temporal non-decreasing vector $\vec{R}=\left(R_{1},R_{2},\ldots ,R_{N}\right)$ following the merged time vector $t_{1}\leq \cdots \leq t_{N}$ , see Figure 1. That is to say, each local maxima $R_{j}$ is, in fact, actual encountered local maxima corresponding to either $X\left(t\right)$ or $Y\left(t\right)$ , or $Z\left(t\right)$ and so on. Finally, the unified limit vector $\left(\eta_{1},\ldots ,\eta_{N}\;\right)$ is introduced with each component $\eta_{j}$ is either $\eta_{X}$ , $\eta_{Y}$ or $\eta_{Z}$ and so on, depending on which of $X\left(t\right)$ or $Y\left(t\right)$ , or $Z\left(t\right)$ *etc.,* corresponds to the current local maxima with the running index j .

Now, the scaling parameter 0<λ≤1 is introduced to, artificially, simultaneously decrease limit values for all response components, namely the new MDOF limit vector $\left(\;\eta_{X}^{\lambda },\eta_{Y}^{\lambda },\eta_{z}^{\lambda },\ldots \right)$ with $\eta_{X}^{\lambda }\equiv \lambda ∙\eta_{X}$ , $\equiv \lambda ∙\eta_{Y}$ , $\eta_{z}^{\lambda }\equiv \lambda ∙\eta_{Z}$ , … is introduced, see Ref. 37. The unified limit vector $\left(\eta_{1}^{\lambda },\ldots ,\eta_{N}^{\lambda }\;\right)$ is introduced with each component $\eta_{j}^{\lambda }$ is either $\eta_{X}^{\lambda }$ , $\eta_{Y}^{\lambda }\;$ or $\eta_{z}^{\lambda }$ and so on. The latter automatically defines probability $P\left(\lambda \right)$ as a function of λ , note that $P\equiv P\left(1\right)$ from Equation (1). Non-exceedance probability $P\left(\lambda \right)$ can be introduced as follows

$$\begin{array}{l} P\left(\lambda \right)=\text{Prob}\left\{R_{N}\leq \eta_{N}^{\lambda },\ldots ,R_{1}\leq \eta_{1}^{\lambda }\right\}\\ \;=\text{Prob}\left\{R_{N}\leq \eta_{N}^{\lambda }\;\right|\;R_{N-1}\leq \eta_{N-1}^{\lambda },\ldots ,R_{1}\leq \eta_{1}^{\lambda }\}\cdot \text{Prob}\left\{R_{N-1}\leq \eta_{N-1}^{\lambda },\ldots ,R_{1}\leq \eta_{1}^{\lambda }\right\}\\ \;=\prod_{j=2}^{N}\text{Prob}\left\{R_{j}\leq \eta_{j}^{\lambda }|\;R_{j-1}\leq \eta_{1j-}^{\lambda },\ldots ,R_{1}\leq \eta_{1}^{\lambda }\right\}\cdot \text{Prob}\left(R_{1}\leq \eta_{1}^{\lambda }\right) \end{array}$$ (2)

Next, cascade of approximations which is based on conditioning is briefly outlined. In practice, ^11^^–^^14^^,^^38^ the dependence between the neighboring $R_{j}$ is not negligible; thus, the following one-step (will be called conditioning level k=1 ) memory approximation is introduced

$$\text{Prob}\left\{R_{j}\leq \eta_{j}^{\lambda }|\;R_{j-1}\leq \eta_{j-1}^{\lambda },\ldots ,R_{1}\leq \eta_{1}^{\lambda }\right\}\approx \text{Prob}\left\{R_{j}\leq \eta_{j}^{\lambda }|\;R_{j-1}\leq \eta_{j-1}^{\lambda }\right\}$$ (3)

for 2≤j≤N (conditioning level k=2 ). The approximation introduced by Equation (3) can be further expressed as

$$\;\text{Prob}\left\{R_{j}\leq \eta_{j}^{\lambda }|\;R_{j-1}\leq \eta_{j-1}^{\lambda },\ldots ,R_{1}\leq \eta_{1}^{\lambda }\right\}\approx \text{Prob}\left\{R_{j}\leq \eta_{j}^{\lambda }|\;R_{j-1}\leq \eta_{j-1}^{\lambda },R_{j-2}\leq \eta_{j-2}^{\lambda }\right\}$$ (4)

where 3≤j≤N (will be called conditioning level k=3 ), and so on. The idea is to monitor each independent failure that happened locally first in time, thus avoiding cascading local inter-correlated exceedances.

Equation (4) exhibits subsequent refinements with respect to the statistical independence assumption. These approximations increasingly accurately approximate statistical dependence between neighboring maxima. Since the original MDOF process $R\left(t\right)$ has been assumed ergodic and thus stationary, the probability $p_{k}\left(\lambda \right)≔\text{Prob}\left\{R_{j}>\eta_{j}^{\lambda }\;\right|\;R_{j-1}\leq \eta_{j-1}^{\lambda },R_{j-k+1}\leq \eta_{j-k+1}^{\lambda }\}$ for j≥k is independent of j , however dependent on the conditioning level k . Thus, non-exceedance probability may be approximated as in the average conditional exceedance rate method, see Refs. 30, 37 for more details on exceedance probability

$$P_{k}\left(\lambda \right)\approx \exp\;\left(-N∙p_{k}\left(\lambda \right)\right),k\geq 1.$$ (5)

Note that Equation (5) follows from Equation (1) if neglecting $\;\text{Prob}\left(R_{1}\leq \eta_{1}^{\lambda }\right)\approx 1$ , as design failure probability must be epsilon order o(1), with N≫k . Equation (5) is analogous to the well-known mean up-crossing rate equation for the stochastic process probability of exceedance. ^10^^,^^37^ There is convergence with respect to k , called here conditioning level

$$P=\lim_{k\to \infty }P_{k}\left(1\right);p\left(\lambda \right)=\lim_{k\to \infty }p_{k}\left(\lambda \right)$$ (6)

Note that Equation (5) for k=1 is equivalent to a well-known non-exceedance probability relationship with the mean up-crossing rate function

$$P\left(\lambda \right)\approx \exp\;\left(-\nu^{+}\left(\lambda \right)\;T\right);\nu^{+}\left(\lambda \right)=\int_{0}^{\infty }\zeta p_{R\dot{R}}\left(\lambda ,\zeta \right)\mathrm{d\zeta}$$ (7)

where $\nu^{+}\left(\lambda \right)$ denotes the mean up-crossing rate of the response level λ for the above assembled non-dimensional vector $R\left(t\right)$ assembled from scaled MDOF system response $\left(\frac{X}{\eta_{X}},\frac{Y}{\eta_{Y}},\frac{Z}{\eta_{Z}},\ldots \right)$ . The mean up-crossing rate is given by the Rice's formula given in Equation (7) with $p_{R\dot{R}}$ being joint probability density for $\left(R,\dot{R}\right)$ with $\dot{R}$ being time derivative $\;R^{\prime }\left(t\right)$ . Equation (7) relies on the Poisson assumption that is up-crossing events of high λ levels (in this paper, it is λ≥1 ) can be assumed to be independent. The latter may not be the case for narrowband responses and higher-level dynamical systems that exhibit cascading failures in different dimensions, subsequent in time, caused by intrinsic inter-dependency between extreme events, manifesting itself in the appearance of highly correlated local maxima clusters within the assembled vector $\vec{R}=\left(R_{1},R_{2},\ldots ,R_{N}\right)$ .

In the above, the stationarity assumption has been used. However, the proposed methodology can also treat the nonstationary case. For nonstationary case, the scattered diagram of m=1,..,M seasonal epidemic conditions, each short-term seasonal state has the probability $\;q_{m}$ , so that $\;\sum_{m=1}^{M}q_{m}=1$ . Next, let one introduce the long-term equation

$$p_{k}\left(\lambda \right)\equiv \sum_{m=1}^{M}p_{k}\left(\lambda ,m\right)q_{m}$$ (8)

with $p_{k}\left(\lambda ,m\right)$ being the same function as in Equation (6) but corresponding to a specific short-term seasonal epidemic state with the number m .

Note that the accuracy of the suggested approach for a large variety of one-dimensional dynamic systems was successfully verified by authors in previous years. ^10^^,^^37^

### Implementing extrapolation method

Introduced by Equation (5) functions $p_{k}\left(\lambda \right)$ are regular in the tail, specifically for values of λ approaching and exceeding 1 . More precisely, for $\lambda \geq \lambda_{0}$ , the distribution tail behaves similar to $\exp\left\{-{\left(\mathrm{a\lambda}+b\right)}^{c}+d\right\}$ with a,b,c,d being suitably fitted constants for suitable tail cut-on $\lambda_{0}\;$ value. Therefore, one can write

$$p_{k}\left(\lambda \right)\approx \exp\left\{-{\left(a_{k}\lambda +b_{k}\right)}^{c_{k}}+d_{k}\right\},\lambda \geq \lambda_{0}\;$$ (9)

Next, by plotting $\ln\left\{\ln\left(p_{k}\left(\lambda \right)\right)-d_{k}\right\}$ versus $\ln\left(a_{k}\lambda +b_{k}\right)$ , often nearly perfectly linear tail behaviour is observed. Optimal values of the parameters $a_{k},b_{k},c_{k},p_{k},q_{k}$ may also be determined applying sequential quadratic programming (SQP) methods, incorporated in NAG Numerical Library. ^39^ Methods described above have been applied as described in methods section. Authors used MATLAB (Mathworks, V 8.6) (RRID:SCR_001622) commercial tool as a basis for their numerical purposes. For more specific author developed code routines, related to the extrapolation method by Equation (9), see ACER. Note that ACER is a repository, containing not only the code, but user manual, examples and references. In this study only extrapolation part of ACER was used. In other words current study presents novel theoretical methodology, but using ACER software previously developed by some of the authors.

### Ethical consideration

Authors confirm that all methods were performed in accordance with the relevant guidelines and regulations according to the Declarations of Helsinki.

## Use case

Methods described in this paper are novel and state of art. Prediction of influenza type epidemics has long been the focus of attention in mathematical biology and epidemiology. It is known that public health dynamics is a seasonally and spatially varying dynamic system that is always challenging to analyse. This section illustrates the efficiency of the above-described methodology using the new method applied to the real-life COVID-19 data sets, presented as a new daily recorded infected patient time series, spread over different regions.

COVID-19 and influenza are contagious diseases with high transmissibility and ability to spread. Seasonal influenza epidemics caused by influenza A and B viruses typically occur annually during winter, presenting a burden on worldwide public health, resulting in around 3–5 million cases of severe illness and 250,000–500,000 deaths worldwide each year, according to the World Health Organization (WHO). ^3^

This section presents a real-life application of the above-described method. The statistical data in the present section are taken from the official website of the National Health Commission of the people's Republic of China. The website provides the number of newly diagnosed cases every day in 13 administrative regions in northern China from 22 January 2020 to 6 April 2022. Patient numbers from thirteen different Chinese administrative regions were chosen as components X,Y,Z,… thus constituting an example of a thirteen dimensional (13D) dynamic biological system.

In order to unify all three measured time series X,Y,Z , the following scaling was performed as follows

$$X\to \frac{X}{\eta_{X}},\;Y\to \frac{Y}{\eta_{Y}},\;Z\to \frac{Z}{\eta_{Z}},\ldots$$ (10)

making all three responses non-dimensional and having the same failure limit equal to 1. Failure limits $\eta_{X},\eta_{Y},\eta_{Z},\ldots$ , or in other words, epidemic thresholds, are not set values and must be decided. The simplest choice would be for different countries to set failure limits equal to the percentage per corresponding country population, making X,Y,Z,… equal to percentage of daily infected per country. In this study, however, twice maxima of daily infected per country have been chosen as failure limits. Note that failure limits may be chosen differently for different dynamic bio-systems. Although the latter choice obviously introduces bias (accumulation point) at λ=0.5 if the number of countrys is large, in this study the number of regions is not that large (below 20 national regions, see Ref. 30 for similar USA study) and proper extrapolation technique may easily circumvent the above mentioned accumulation point bias.

Next, all local maxima from three measured time series were merged into one single time series by keeping them in time non-decreasing order: $\vec{R}=\left(\max\left\{X_{1},Y_{1},Z_{1},\ldots \right\},\ldots ,\max\left\{X_{N},Y_{N},Z_{N},\ldots \right\}\right)$ with the whole $\vec{R}\;$ vector being sorted according to non-decreasing times of occurrence of these local maxima.

**Figure 2.**
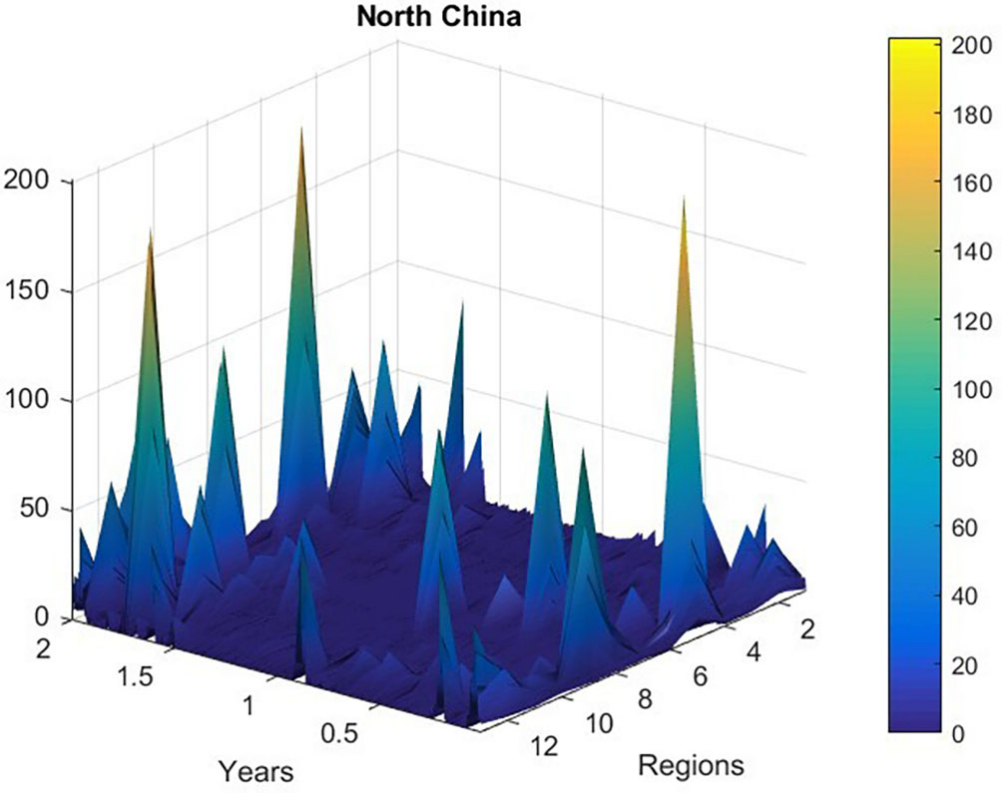
New daily recorded patients number plotted as a 2D time-space surface, data was according to http://www.nhc.gov.cn.

**Figure 3.**
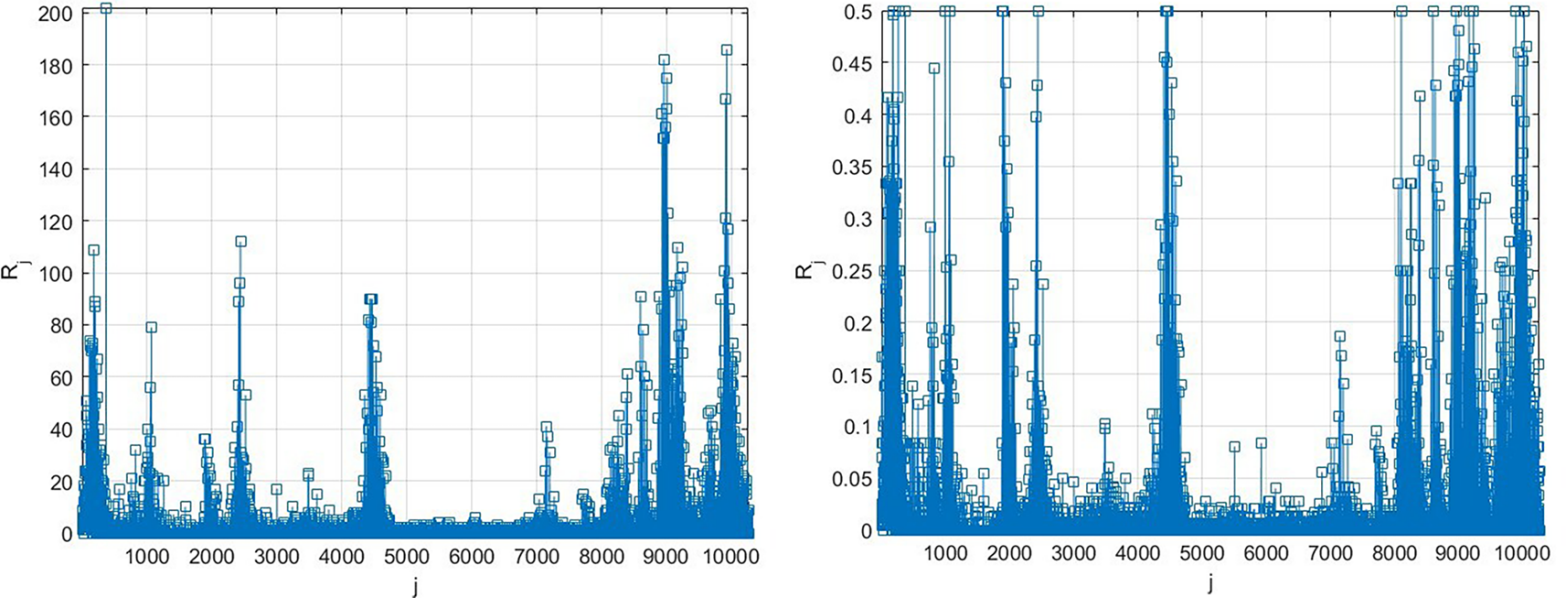
Number of new daily recorded patients as 13D vector $\vec{R}$ . Left: as it is, Right: scaled by Equation (10).

Figure 2 presents new daily recorded patients number plotted as a time-space 2D surface using MATLAB. Figure 3 presents the number of new daily recorded patients as a 13D vector $\vec{R}$ , consisting of assembled regional new daily patient numbers. Note that vector $\vec{R}$ does not have physical meaning on its own, as it is assembled of different regional components with different epidemic backgrounds. Index j is just a running index of local maxima encountered in a non-decreasing time sequence.

**Figure 4.**
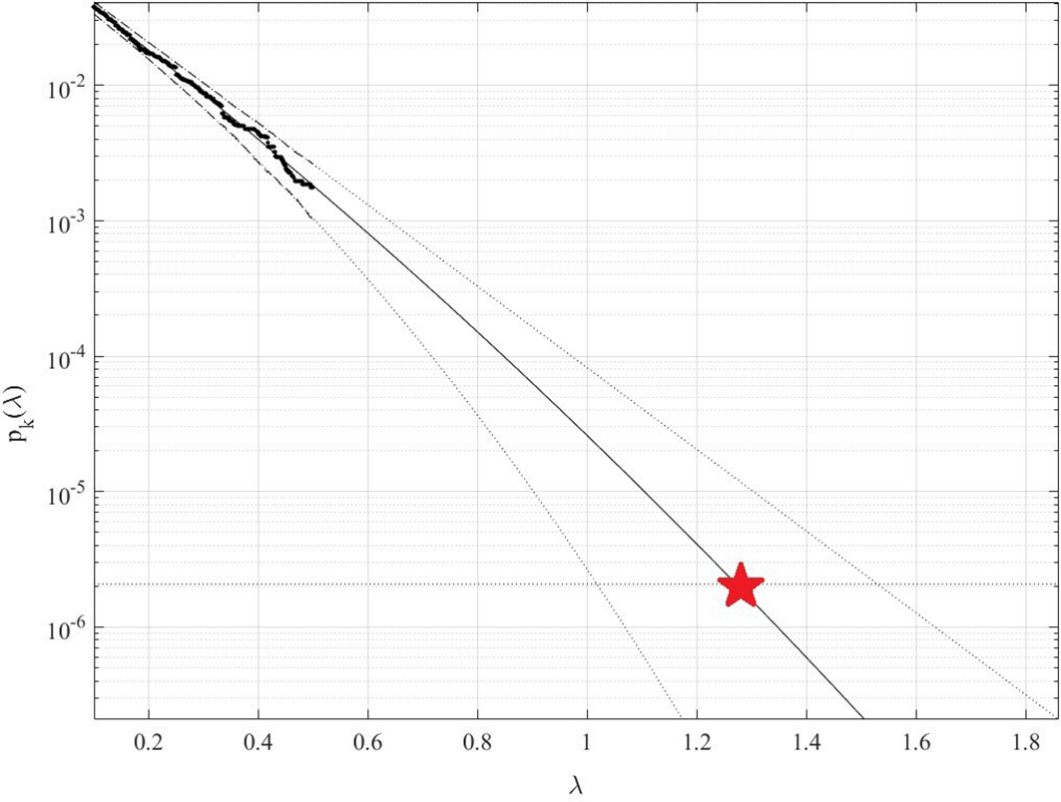
100 years return level (horizontal dotted line) extrapolation of $p_{k}\left(\lambda \right)$ towards critical (system failure) level (indicated by star) and beyond. Extrapolated 95% CI indicated by dotted lines, using https://github.com/cran/acer.

Figure 4 presents 100 years return level extrapolation according to Equation (9) towards epidemic outbreak with 100 year return period, indicated by the horizontal dotted line, and somewhat beyond, λ=0.1 cut-on value was used. Dotted lines indicate extrapolated 95% confidence interval according to Equation (10). According to Equation (5) $p\left(\lambda \right)$ is directly related to the target failure probability 1−P from Equation (1). Therefore, in agreement with Equation (5), system failure probability $1-P\approx 1-P_{k}\left(1\right)$ can be estimated. Note that in Equation (5), N corresponds to the total number of local maxima in the unified response vector $\;\vec{R}$ .Conditioning parameter k=3 was found to be sufficient due to occurrence of convergence with respect to k , see Equation (6). Figure 4 exhibits reasonably narrow 95% CI. The latter is an advantage of the proposed method.

Note that while being novel, the above-described methodology has a clear advantage of utilizing available measured data set quite efficiently due to its ability to treat health system multi-dimensionality and perform accurate extrapolation based on quite limited data set. Note that, predicted non-dimensional λ level, indicated by star in Figure 4, represents probability of epidemic outbreak at any northern province in China in the years to come.

## Discussion

Traditional health bio-systems reliability methods dealing with observed/measured time series do not have the ability to efficiently deal with high dimensionality and cross-correlation between different system responses. The main advantage of the introduced methodology is its ability to study high dimensional non-linear dynamic systems reliability.

Despite the simplicity, the present study successfully offers a novel multidimensional modelling strategy and a methodological avenue to implement the forecasting of an epidemic during its course, if it is assumed to be stationary in time. Proper setting of epidemiological alarm limits (failure limits) per province has been discussed, see Section Use case.

This paper studied recorded COVID-19 patient numbers from thirteen different Chinese northern provinces, constituting an example of a thirteen dimensional (13D) and ten-dimensional (10D) dynamic biological system respectively observed in 2020-2022. The novel reliability method was applied to new daily patient numbers as a multidimensional system in real-time. ^30^

The main conclusion is that if the public health system under local environmental and epidemiologic conditions in northern China is well managed. Predicted 100 year return period risk level λ of epidemic outbreak is reasonably low. However, there is an ultra-low risk of a future epidemic outbreak in both countries, at least in 100 years horizon.

This study outlines a general-purpose, robust and straightforward multidimensional bio-system reliability method. The method introduced in this study has been previously successfully validated by application to a wide range of engineering models, ^30^^,^^40^^–^^49^ but only for one and two-dimensional system responses and, in general, very accurate predictions were obtained. Both measured and simulated time series responses can be analysed using the proposed method. It is shown that the method produced an acceptable 95% confidence interval, see Figure 4. Thus, the suggested methodology may be used as a tool in various non-linear dynamic biological systems reliability studies. The presented COVID-19 example does not limit potential areas of new method applicability by any means.

## Data availability

The datasets analyzed during the current study are publicly available from the daily recorded patients dataset in China during 2020-2022 years, are available at http://www.nhc.gov.cn/. Daily recorded patients data was structured per province and per calendar day, namely it was straightforward to extract and systematize joint statistical distribution as a function of both space and time.

## Software availability

Source code along with demo and user manual and examples used for extrapolation available from: ACER this third-party software is under license GPL-3.
