# Supplementary material for: Gaidai reliability method for long-term coronavirus modelling
Source: F1000Res. 2023 Nov 21;11:1282. Originally published 2022 Nov 10. [Version 3] doi: 10.12688/f1000research.125924.3 (PMC10728585; doi:10.12688/f1000research.125924.3)
Supplement: Peer review history

# Reviewer report: Reviewer response for version 3

**DOI:** 10.5256/f1000research.158999.r242811

**Published:** 2024-04-01

**Authors:** Eduard Campillo-Funollet

**Affiliations:** 1 Mathematics and Statistics, Lancaster University, Lancaster, England, UK

The manuscript presents the application of an extreme-value methodology to the analysis of epidemiological data. The methodology have been applied in the past to other types of data. The manuscript illustrates the methodology using data from the COVID-19 pandemic in China.

 I have several reservations about the manuscript, in particular regarding the technical background and the conclusions.

 Major concerns:

 - The claim that this study "fully space-time dynamic biosystem" (Introduction, paragraph beginning with "Biological systems...") is not justified. If anything, the proposed method does not account for the dynamics of the disease, and in fact it relies on the assumption of stationary data.

 - Similarly, the assumption of the process being stationary is a strong assumption in the context of an evolving epidemic. It is mentioned briefly but its implications should be discussed.

 - The description of the methods mixes using three components, with using many. See for example the paragraph before equation 2. The presentation would be clearer if the notation was adapted to use a generic number of components n, instead of using three and saying "etcetera", for exapmle in "X,Y,Z,...". This also affects the rest of the equations in the presentaiton, for example \eta_j is not clearly defined in (3).

 - The assumption of an exponential tail ("exceedance probability", eq. 6) should be clearly justified in paper, rather than citing an application paper.

 - The 100-year return level in Fig 4, which summarises very well the applicability of the method, should be discussed in detail. The data is from a two year period during the COVID-19 pandemic, and the method essentially captures the peaks of new cases during that time. However, the changes in the population (e.g. immunity or vaccinations) make the past data not representative of the current or future situations, and from an epidemiological perspective, make the 100-year return level difficult to apply in practice. Could the methodology be applied in shorter time frames, maybe even allowing for some validation of the method?

 Other minor comments:

 - Several references are not relevant to the paper For example, 4-11 are cited as related to epidemics, and in fact they are about renewable energy, wind turbines and other topics. Similarly with 14, 15.

 - Several citation styles are used in the paper (see for example the first paragraph of the introduction)

Is the rationale for developing the new method (or application) clearly explained?

Partly

Is the description of the method technically sound?

Partly

Are the conclusions about the method and its performance adequately supported by the findings presented in the article?

No

If any results are presented, are all the source data underlying the results available to ensure full reproducibility?

Yes

Are sufficient details provided to allow replication of the method development and its use by others?

Yes

Reviewer Expertise:

Epidemiology, statistics

I confirm that I have read this submission and believe that I have an appropriate level of expertise to confirm that it is of an acceptable scientific standard, however I have significant reservations, as outlined above.

# Reviewer report: Reviewer response for version 3

**DOI:** 10.5256/f1000research.158999.r226132

**Published:** 2023-12-18

**Authors:** Md. Ashraful Babu

**Affiliations:** 1 International University of Business Agriculture and Technology, Dhaka, Dhaka Division, Bangladesh; 2 Department of Physical Sciences, Independent University, Bangladesh, Dhaka, Dhaka Division, Bangladesh

The article presents a new method for long-term coronavirus forecasting using statistical extreme value theory and MATLAB optimization. The rationale for this method is clearly explained, addressing the limitations of existing models in varying epidemic scenarios. While the description of the method is technically sound, its reproducibility is partly limited due to insufficient detailed procedural and algorithmic information. Additionally, the full reproducibility of results is constrained as the underlying source data is not entirely available. The conclusions drawn about the method's performance are well-supported by the findings.

 To enhance the scientific soundness and utility of the article, the authors should consider:

1. Providing more detailed procedural and algorithmic descriptions, enabling others to replicate the method more accurately.
2. Including or making accessible the complete source data used in their study, allowing for full validation and replication of results.

Addressing these points will significantly improve the paper's comprehensiveness and its applicability in different research contexts.

Is the rationale for developing the new method (or application) clearly explained?

Yes

Is the description of the method technically sound?

Yes

Are the conclusions about the method and its performance adequately supported by the findings presented in the article?

Yes

If any results are presented, are all the source data underlying the results available to ensure full reproducibility?

Partly

Are sufficient details provided to allow replication of the method development and its use by others?

Partly

Reviewer Expertise:

Operations Research, Machine Learning, Deep Learning, Optimization, TIme Series Forecasting

I confirm that I have read this submission and believe that I have an appropriate level of expertise to confirm that it is of an acceptable scientific standard, however I have significant reservations, as outlined above.

# Reviewer report: Reviewer response for version 2

**DOI:** 10.5256/f1000research.144806.r174507

**Published:** 2023-11-14

**Authors:** Adnan Khan

**Affiliations:** 1 Department of Mathematics, Lahore University of Management Sciences, Lahore, Pakistan

The authors have addressed many of the language and formatting concerns. There remain a few language issues in that the construct is rather cumbersome. For example

*"This section presents novel bio-system reliability method, presently invented by authors and previously not existing. Advocated methodology used generalized extreme value (GEV) theory as a basis for its buildup.*" 

 However, while not ideal in my view, the paper can be understood in its current form. 

 My concern regarding the method still remains, it seems that all work using this technique has involved one of the authors of the current work. This fact is of some concern to me.

Is the rationale for developing the new method (or application) clearly explained?

No

Is the description of the method technically sound?

Partly

Are the conclusions about the method and its performance adequately supported by the findings presented in the article?

No

If any results are presented, are all the source data underlying the results available to ensure full reproducibility?

Yes

Are sufficient details provided to allow replication of the method development and its use by others?

No

Reviewer Expertise:

Mathematical Biology

I confirm that I have read this submission and believe that I have an appropriate level of expertise to confirm that it is of an acceptable scientific standard, however I have significant reservations, as outlined above.

# Response

**Published:** 2023-11-15

**Authors:** Oleg Gaidai

**Affiliations:** shanghai ocean university, China

*"This section presents novel bio-system reliability method, presently invented by authors and previously not existing. Advocated methodology used generalized extreme value (GEV) theory as a basis for its buildup.*" 

**Answer:** thanks, passages corrected.

 However, while not ideal in my view, the paper can be understood in its current form. 

 My concern regarding the method still remains, it seems that all work using this technique has involved one of the authors of the current work. This fact is of some concern to me.

**Answer:** thanks, now manuscript and method title modified to stress authorship of Oleg Gaidai (the leading author).

# Reviewer report: Reviewer response for version 1

**DOI:** 10.5256/f1000research.138279.r157163

**Published:** 2023-02-21

**Authors:** Adnan Khan

**Affiliations:** 1 Department of Mathematics, Lahore University of Management Sciences, Lahore, Pakistan

The authors want to propose a new methodology to make reliable long term forecast of COVID-19 infection rates, in particular they want to consider multidimensional data that may have cross correlations.  

 Comments:

 The paper is not well written and very hard to read. There are grammatical errors as well as sentences which are very unclear to me. A few examples from the first section are given below:

- "The latter is usually due to many degrees of system freedom and random variables governing dynamic biological systems, spread over extensive terrain." (Hard to understand)
- "For example, Gumbel used EVT to estimate the demographic of various populations in." (Incomplete sentence)
- "Moreover, a specific non-dimensional factor λλ is introduced to predict the latter epidemic risk at any time and any place." (What exactly is the biological meaning of this parameter, this seems to be a critical component of the study and really needs a lot of detail when it is introduced.)

Many of the equations are not well explained e.g. Eq 2 third equality has a typo ‘ ’, similarly equation 5 needs to be explained further.

 In the discussion section there is a mention of ‘both countries’, not clear which country besides China is being referred to.

 These are just some examples of unclear text, hence my recommendation below.

 Recommendation:

 The aims and results are not well explained. My recommendation is that the authors may want to extensively edit the paper, making their goals, method and results clearer and the paper more readable.

Is the rationale for developing the new method (or application) clearly explained?

No

Is the description of the method technically sound?

Partly

Are the conclusions about the method and its performance adequately supported by the findings presented in the article?

No

If any results are presented, are all the source data underlying the results available to ensure full reproducibility?

Yes

Are sufficient details provided to allow replication of the method development and its use by others?

No

Reviewer Expertise:

Mathematical Biology

I confirm that I have read this submission and believe that I have an appropriate level of expertise to state that I do not consider it to be of an acceptable scientific standard, for reasons outlined above.

# Response

**Published:** 2023-02-23

**Authors:** Oleg Gaidai

**Affiliations:** shanghai ocean university, China

**Response to reviewer comments**

 Note:  since publication time of this methodology in 2022, a number of other applications of this method has been published, to mention few:

 1) Gaidai, O., Cao, Y., Loginov, S., 2023, "Global cardiovascular diseases death rate prediction", Current Problems in Cardiology, https://doi.org/10.1016/j.cpcardiol.2023.101622

 2) Gaidai, O., Cao, Y.,  Xing, Y., Balakrishna, R., 2023, "Extreme springing response statistics of a tethered platform by deconvolution", International Journal of Naval Architecture and Ocean Engineering, https://doi.org/10.1016/j.ijnaoe.2023.100515

 3) Gaidai, O., Xing, Y., Balakrishna, R., Xu, J., 2023,"Improving extreme offshore wind speed prediction by using deconvolution", Heliyon, https://doi.org/10.1016/j.heliyon.2023.e13533

 4) Gaidai, O., Xing, Y., 2023,"Prediction of death rates for cardiovascular diseases and cancers", Cancer Innovation, http://doi.org/10.1002/cai2.47

 5) Gaidai, O., Wang F., Yakimov, V., 2023, "COVID-19 multi-state epidemic forecast in India", Proceedings of the Indian National Science Academy, DOI: 10.1007/s43538-022-00147-5 

***Making proposed method well validated***, reference list is updated now. Equations now restructured, so that all equations are collected only in one Section.

 The paper is not well written and very hard to read. There are grammatical errors as well as sentences which are very unclear to me. A few examples from the first section are given below:

 "The latter is usually due to many degrees of system freedom and random variables governing dynamic biological systems, spread over extensive terrain." (Hard to understand)

 Answer: Changed to:  “The latter is usually due to the fact that dynamic biological and environmental systems possess high number of degrees of freedom (in other words dimensional components), as well as system dependency on location – making bio-system of interest spatio-temporal.”

 "For example, Gumbel used EVT to estimate the demographic of various populations in." (Incomplete sentence)

  Answer: Sentence removed. 

 "Moreover, a specific non-dimensional factor λ is introduced to predict the latter epidemic risk at any time and any place." (What exactly is the biological meaning of this parameter, this seems to be a critical component of the study and really needs a lot of detail when it is introduced.)

 Answer: Changed to:  “introduced to **unify** various bio-system components having different failure limits”. As seen later in the text  λ being just a non-dimensional parameter, used to indicate system failure, namely when λ reaches 1.

 Many of the equations are not well explained e.g. Eq 2 third equality has a typo ‘’, similarly equation 5 needs to be explained further.

 Answer: Equation corrected, now its Eq. (3).

 In the discussion section there is a mention of ‘both countries’, not clear which country besides China is being referred to.

 Answer: Thanks a lot for your valuable comments, changed now to a “chosen country of interest”

 Recommendation:

 The aims and results are not well explained. My recommendation is that the authors may want to extensively edit the paper, making their goals, method and results clearer and the paper more readable.

 Answer: Text and equations modified. In a view of recent publication on this method authors now refer to already published papers: 1)-5) (see above).

# Reviewer report: Reviewer response for version 1

**DOI:** 10.5256/f1000research.138279.r155545

**Published:** 2022-11-29

**Authors:** Oluwakemi E Abiodun

**Affiliations:** 1 Department of Physical Sciences, Landmark University, Omu-Aran, Nigeria

This study came up with a new way to look at the dependability of biosystems. This method was tested for a long enough time to make a credible long-term prediction of the probability of the extreme new coronavirus mortality rate. This methodology was applicable to the environmental and health systems in numerous nations. Provinces that were examined estimated their future maximum patient populations. This study introduces a method for assessing the dependability of multidimensional biosystems. The suggested methodology can be utilized as a tool in a variety of non-linear, dynamic biological systems reliability studies.

 Oleg and his colleagues come up with the MDOF (multiple degrees of freedom) health response vector process. They do this in a new way. In addition, a novel new statistical approach—a generalized extreme value (GEV)—that was directly applied to unprocessed clinical data and is capable of dealing with territorial mapping was used as the foundation for model construction. This part of the study gives future researchers a new idea about how they might try to predict how an outbreak will change over time.

 This paper is, in my opinion, an excellent contribution to the literature.

Is the rationale for developing the new method (or application) clearly explained?

Yes

Is the description of the method technically sound?

Yes

Are the conclusions about the method and its performance adequately supported by the findings presented in the article?

Yes

If any results are presented, are all the source data underlying the results available to ensure full reproducibility?

Partly

Are sufficient details provided to allow replication of the method development and its use by others?

Yes

Reviewer Expertise:

Mathematical and statistical modeling of infectious disease

I confirm that I have read this submission and believe that I have an appropriate level of expertise to confirm that it is of an acceptable scientific standard.
